# *JAMA* Published Fewer Industry-Funded Studies after Introducing a Requirement for Independent Statistical Analysis

**DOI:** 10.1371/journal.pone.0013591

**Published:** 2010-10-22

**Authors:** Elizabeth Wager, Rahul Mhaskar, Stephanie Warburton, Benjamin Djulbegovic

**Affiliations:** 1 Sideview, Princes Risborough, Buckinghamshire, United Kingdom; 2 Center for Evidence-based Medicine and Health Outcomes Research, University of South Florida, Tampa, Florida, United States of America; 3 H. Lee Moffitt Cancer Center & Research Institute, Tampa, Florida, United States of America; Swiss Paraplegic Research, Switzerland

## Abstract

**Background:**

*JAMA* introduced a requirement for independent statistical analysis for industry-funded trials in July 2005. We wanted to see whether this policy affected the number of industry-funded trials published by *JAMA*.

**Methods and Findings:**

We undertook a retrospective, before-and-after study of published papers. Two investigators independently extracted data from all issues of *JAMA* published between 1 July 2002 and 30 June 2008 (i.e., three years before and after the policy). They were not blinded to publication date. The randomized controlled trials (RCTs) were classified as industry funded (IF), joint industry/non-commercial funding (J), industry supported (IS) (when manufacturers provided materials only), non-commercial (N) or funding not stated (NS). Findings were compared and discrepancies resolved by discussion or further analysis of the reports. RCTs published in *The Lancet* and *NEJM* over the same period were used as a control group. Between July 2002 and July 2008, *JAMA* published 1,314 papers, of which 311 were RCTs. The number of industry studies (IF, J or IS) fell significantly after the policy (p = 0.02) especially for categories J and IS. However, over the same period, the number of industry studies rose in both *The Lancet* and *NEJM*.

**Conclusions:**

After the requirement for independent statistical analysis for industry-funded studies, *JAMA* published significantly fewer RCTs and significantly fewer industry-funded RCTs. This pattern was not seen in the control journals. This suggests the *JAMA* policy affected the number of submissions, the acceptance rate, or both. Without analysing the submissions, we cannot check these hypotheses but, assuming the number of published papers is related to the number submitted, our findings suggest that *JAMA'*s policy may have resulted in a significant reduction in the number of industry-sponsored trials it received and published.

## Introduction

In July 2005, *JAMA* (the Journal of the American Medical Association) introduced a requirement that industry-sponsored studies must undergo independent analysis by statisticians at academic institutions [1]. This policy has not been adopted by other medical journals and, in fact, has been criticised by some statisticians [2]. When the policy was introduced, it was rumoured that some pharmaceutical companies would boycott *JAMA* and refuse to submit papers there. We therefore wanted to see whether the requirement for independent statistical analysis was associated with any change in the number of industry-sponsored studies published in *JAMA*. We therefore compared the number of industry-sponsored studies published in *JAMA* before and after the policy was introduced and used papers published in two other high impact, general, medical journals as a control.

## Methods

Two investigators independently coded all randomized controlled trials (RCTs) published in *JAMA* from 1 July 2002 to 30 June 2008 (i.e., 3 years before and after the policy was introduced). The investigators were not blinded to publication date. RCTs were identified from article titles (in the journal's electronic tables of contents) and inspection of the methods (if in doubt). Electronic versions (PDFs) of all RCTs were downloaded from the journal's website and their funding determined. Trials were classified as: industry funded (IF), joint industry plus non-commercial funding (J), industry supported (IS) (when manufacturers provided materials only but had no input into research design or execution), non-commercial (N) or funding not stated (NS). For the statistical analysis, RCTs were classified *a priori* as ‘Industry’ if they had any commercial funding or support (i.e., we considered all those in the IF, J and IS categories as ‘Industry’ studies). RCTs published in *The Lancet* and *New England Journal of Medicine (NEJM)* over the same period provided the control (as these journals did not change their requirements). The same methods of retrieval and categorization were used for *JAMA* and the control journals. In all cases, discrepancies were resolved by discussion or further analysis until consensus was achieved.

## Results

**Figure 1:**
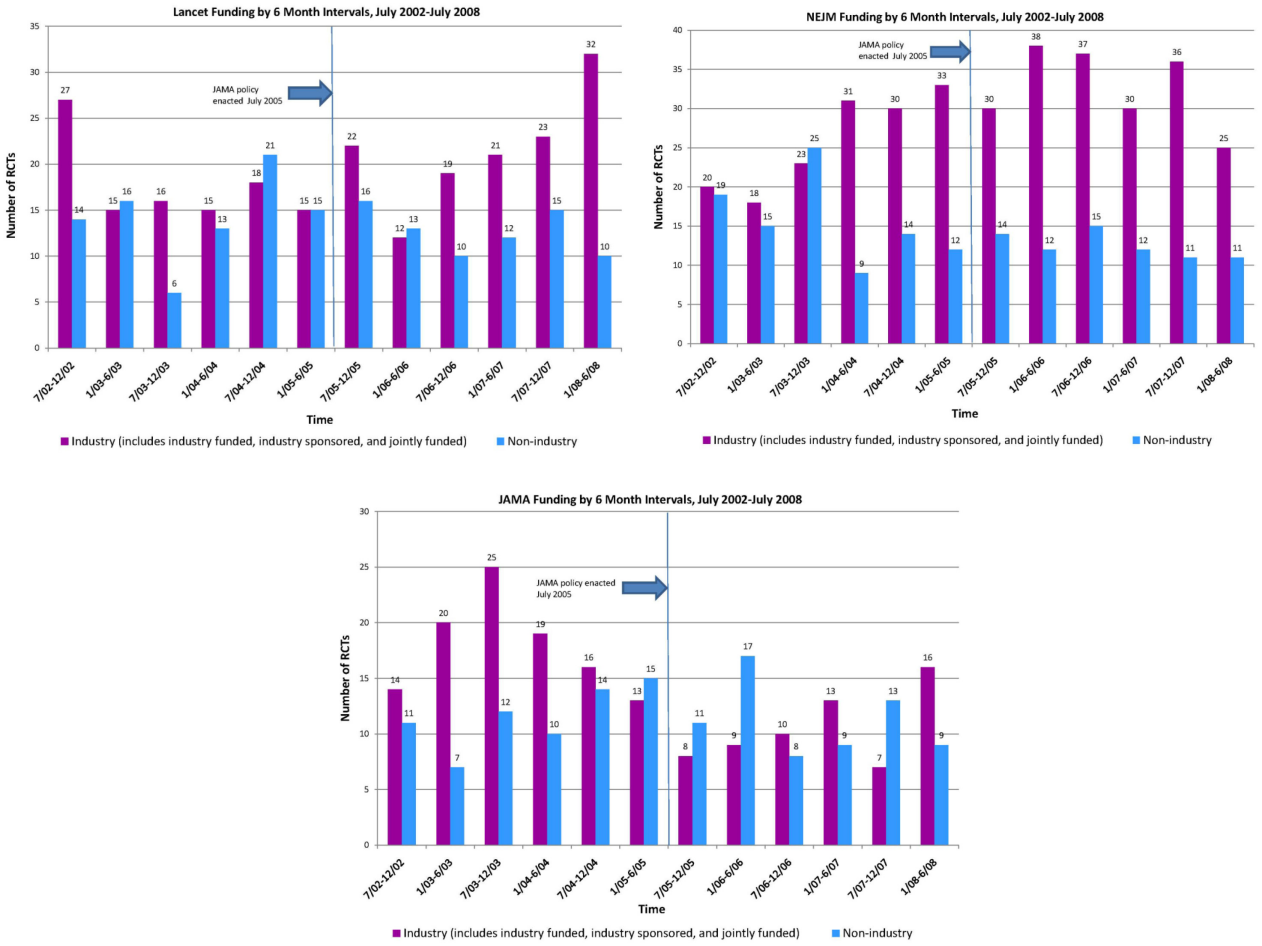
Number of randomized controlled trials published by (a) *JAMA*, (b) *NEJM* and (c) *Lancet* by funding source over time. (*JAMA* introduced its policy for independent statistical analysis for industry-funded studies in July 2005.) ‘Industry’ funded trials includes those that were: solely industry funded; jointly funded by industry and non-commercial sources; or supported by the industry (i.e. the manufacturer provided materials only).

The total number of RCTs published in *JAMA* decreased significantly after July 2005 but increased in *NEJM* and remained stable in *The Lancet* (Table 1, Figure 1). The proportion of Industry RCTs also decreased significantly in *JAMA*, but rose significantly in both *NEJM* and *The Lancet* over the same period.

**Table 1:** Number of randomized trials (RCTs) and studies with commercial funding published in *JAMA, NEJM* and *The Lancet* in the 3 years before and after JAMA's policy on independent statistical analysis (July 2005).

	*JAMA*		*NEJM*		*Lancet*	
	Before^1^	After^2^	Before^1^	After^2^	Before^1^	After^2^
All papers	677	637	616	606	530	529
RCTs (%)	178 (26)	133 (21)	258 (42)	276 (46)	199 (38)	210 (40)
p-value	0.02		0.20		0.48	
Industry^3^ (%)	107 (63)	63 (37)	155 (44)	196 (56)	106 (45)	129 (55)
p-value	0.01		0.03		0.01	
IF, IS, J^4^	49, 35, 23	39, 19, 5	82, 26, 47	102, 44, 50	47, 30, 29	79, 18, 32
RCTs with funding not stated/unclear	2	3	10	4	7	6

1 1 July 2002–30 June 2005.

2 1 July 2005–30 June 2008.

3 Studies with any commercial funding.

4 IF  =  solely industry funded, IS  =  industry support (drugs or equipment only), J  =  jointly funded by industry and non-commercial sources.

Further categorization to distinguish trials funded completely by industry (IF) from those with joint funding (J) or those for which companies provided support (i.e. drugs or equipment) but took no part in design or analysis (IS) showed a more complex pattern. All categories of Industry studies (IF, IS and J) decreased in *JAMA*, while in *NEJM*, IF and IS increased while J remained stable, and in *Lancet* IF increased markedly but IS decreased and J remained stable (Table 1).

The number of reports with no funding source stated, or for which the investigators, despite their experience in the area, had difficulty determining the nature of the funding source was notable (32 articles altogether in the three journals) although the actual proportion was small (2.6%). While caution should be exercised in interpreting such small numbers, it appeared that *JAMA* had a lower proportion of RCTs with unstated or unclear funding than the other journals (5 in *JAMA*, 14 in *NEJM*, 13 in *Lancet*).

Although *JAMA* announced its policy in July 2005, we could not tell exactly when it started to take effect (since several months usually elapse between submission and publication of articles). We therefore conducted a sensitivity analysis to measure the effect of the articles published between July and December 2005. During this (possible transition) period, *JAMA* published 19 RCTs. If these are excluded from the analysis, our findings are unchanged and we still observe significantly fewer Industry RCTs in the period following the new policy (p = 0.02).

## Discussion

The proportion of industry-sponsored studies published in *JAMA* decreased significantly after the journal required independent statistical analysis but increased in the two control journals over the same period. While our study cannot explain the underlying causes, we suggest that the most plausible explanation for our findings is that there is a finite number of high-impact general journals in which industry wants to publish RCTs so a policy in one journal may result in a ‘zero-sum game’: if fewer RCTs are published in *JAMA* then more are published in *NEJM* and *Lancet* (or elsewhere). However, our findings may, instead, be due to other causes, such as policies at *Lancet* and/or *NEJM* to publish more industry-funded studies, or other more general trends in funding patterns or publication affecting the proportion of industry-funded studies being published. One surprising aspect of our findings was that the number of industry-supported studies and jointly funded studies published by *JAMA* decreased to a similar extent as solely industry-funded studies, despite the fact that such studies are generally analysed by academic statisticians and might therefore be expected to be unaffected by the policy requirements.

Since we could analyse only published trials, we cannot tell whether the policy affected submission or acceptance rates. However, according to the journal, the total number of submissions to *JAMA* rose steadily between 2002 and 2005 (from 4615 to 5744 per year) then fell in 2006 to 5354, rose to 5551 in 2007 and fell slightly to 5525 in 2008 [3].

We requested information from *JAMA* on acceptance rates according to funding source but were informed that these were not available. However, it seems reasonable to assume that the number of published papers is probably related to the number submitted, and, given the overall fall in the number of submissions, and the rumours of an industry ‘boycott’ of *JAMA*, this is consistent with a decrease in the number of industry-sponsored trials being submitted to *JAMA* at least in the years immediately following the introduction of the policy.

*JAMA'*s policy has not been adopted by other major journals and has been criticised by some statisticians [2]. We did not attempt to measure the quality of the published papers (and, in particular their statistical methods) to see if this had changed. It would also be interesting to discover what, if any, changes, the independent statistical reviewers suggested.

The key question is whether *JAMA*'s policy protected its readers by publishing more reliable trial reports. We cannot tell if the policy has improved the standard of reporting but it was associated with a significant decrease in the number of industry-sponsored randomized trials published by *JAMA* which was not observed in two other high-ranking general medical journals. To the extent that one associates industry sponsorship with increased risk for biased reporting [4] one can argue that the policy may have achieved its desired results at least within *JAMA*. However, if it simply had the effect of deflecting reports of industry-funded studies from a journal with a more-demanding policy (ie *JAMA*) to journals with less-demanding policies, then the overall effect on the literature would have been minimal.
